# Identification of a Pyroptosis-Related Prognostic Signature Combined With Experiments in Hepatocellular Carcinoma

**DOI:** 10.3389/fmolb.2022.822503

**Published:** 2022-03-04

**Authors:** Huihui Li, Tang Li, Xiaohua Zhang

**Affiliations:** ^1^ Department of Thyroid and Breast Surgery, The First Affiliated Hospital of Wenzhou Medical University, Wenzhou, China; ^2^ Department of Respiratory and Critical Care Medicine, The Affiliated People’s Hospital to Ningbo University, Ningbo, China

**Keywords:** HCC, pyroptosis, gene-signature, *in-vitro* experiment, SCAF11

## Abstract

Hepatocellular carcinoma (HCC) is one of the most common malignancies worldwide with poor prognosis. There is a necessary search for improvement in diagnosis and treatment methods to improve the prognosis. Some useful prognostic markers of HCC are still lacking. Pyroptosis is a type of programmed cell death caused by the inflammasome. It is still unknown whether pyroptosis-related genes (PRGs) are involved in the prognosis in HCC. The gene expression and clinical data of LIHC (liver hepatocellular carcinoma) patients were downloaded from The Cancer Genome Atlas (TCGA) and the International Cancer Genome Consortium database (ICGC). In this study, we identified 40 PRGs that were differentially expressed between LIHC and normal liver tissues. Based on the TCGA-LIHC cohort, a 9-gene prediction model was established with the Least absolute shrinkage and selection operator (LASSO)-penalized Cox regression. The risk score was calculated according to the model in the TCGA-LIHC cohort and the ICGC-LIHC cohort. Utilizing the median risk score from the TCGA cohort, LIHC patients from the ICGC-LIHC cohort were divided into two risk subgroups. The Kaplan–Meier (KM) survival curves demonstrated that patients with lower risk scores had significantly favorable overall survival (OS). Combined with the clinical characteristics, the risk score was an independent factor for predicting the OS of LIHC patients in both the TCGA-LIHC cohort and the ICGC-LIHC cohort. Functional enrichment and immune function analysis were carried out. Furthermore, a nomogram based on risk score, age, gender, and tumor stage was used to predict mortality of patients with LIHC. Moreover, KM survival analysis was performed for 9 genes in the risk model, among which CHMP4A, SCAF11, and GSDMC had significantly different results and the ceRNA network was constructed. Based on the core role of SCAF11, we performed loss-of-function experiments to explore the function of SCAF11 *in vitro*. Suppression of SCAF11 expression inhibited the proliferation, attenuated the migration and invasion, and induced apoptosis of liver cancer cell lines. In conclusion, the pyroptosis-related model and nomogram can be utilized for the clinical prognostic prediction in LIHC. This study has demonstrated for the first time that SCAF11 promotes the progression of liver cancer.

## Introduction

Hepatocellular carcinoma (HCC) is the main cause of cancer-associated death worldwide and has high morbidity and mortality (Piñero et al., 2020; Rebouissou and Nault, 2020; Pinter et al., 2021). Despite advancements in therapies, the overall prognosis for HCC remains poor (Umeda et al., 2019; Kumari et al., 2021). Additionally, useful prognostic markers of HCC are still unavailable. Moreover, due to individual differences, patients with similar tumor stages or pathologic structures may have distinctly different prognosis (Umeda et al., 2019; Pinter et al., 2021). Therefore, it is important to explore new and reliable biomarkers for predicting the prognosis of HCC.

As a kind of programmed cell death caused by inflammasomes, pyroptosis results in the cleavage of gasdermin D (GSDMD) and activation of cytokines (Bergsbaken et al., 2009; Ju X. et al., 2021) with features such as cellular membrane pore generation, cytoplasmic swelling, membrane rupture, and release of cytoplasmic cytokines such as IL-18 and IL-1β into the extracellular environment (Zheng and Li, 2020). Pyroptosis can be categorized in two distinct ways: caspase-1-dependent classical inflammasome signaling and caspase-4/5/11-dependent nonclassical inflammasome signaling (Fang et al., 2020). The key protein in both signaling pathways is GSDMD. In classical pyroptosis, many pathogen-related molecular patterns or danger-related molecular patterns, such as bacterial peptidoglycans, heat shock proteins, and damaged mitochondrial DNA, activate the inflammasome, such as the nucleotide-binding oligomerization domain-like receptor (NLR), and are absent in melanoma 2 (AIM2) (Wang et al., 2019; Ruan et al., 2020). Furthermore, the process leads to the recruitment of caspase-1 protein into the inflammasome complex. The inflammasome complex is composed of the sensor, adaptor-like apoptosis-related speck-like proteins (ASCs), and caspase activation and recruitment domain (CARD) of ASCs (Bergsbaken et al., 2009). The activated complex can result in the secretion of IL-18 and IL-1β. In addition, activated caspase-1 cleaves GSDMD into two sections, including the C-terminal and N-terminal domains. The N-terminal domain can cause the formation of cellular membrane pores. These pores facilitate cell swelling, membrane rupture, and the release of inflammatory cytokines and ultimately lead to pyroptosis (Zheng et al., 2020). According to recent studies, pyroptosis has been linked to the pathophysiological processes of many diseases, including tumors, inflammatory diseases, and immune diseases (Hu et al., 2010; Zaki et al., 2011; Lamkanfi and Dixit, 2012; Zhou et al., 2020). At present, there are few studies about pyroptosis in HCC. Whether these pyroptosis-related genes (PRGs) affect the prognosis of patients with HCC is still unclear.

Based on the above findings, we first established a PRG-related model on the basis of mRNA expression and clinical data of liver hepatocellular carcinoma (LIHC) patients from the TCGA dataset and validated it in the ICGC cohort. We investigated the function and difference in immune cells between different risk subgroups classified by the model. Furthermore, a KM survival analysis was respectively conducted for 9 genes of the risk model, among which CHMP4A, SCAF11, and GSDMC had significantly different results, and the ceRNA network of pyroptosis in HCC was constructed. Finally, based on the core role of SCAF11, we performed loss-of-function experiments to study the function of SCAF11 *in vitro*.

## Methods

### Data Collection and Processing

The mRNA expression and clinical information of LIHC were downloaded from TCGA (https://portal.gdc.cancer.gov/). We obtained 375 tumor samples and 50 normal samples with expression and clinical data. The normalization of expression data was performed with the log2 (TPM+1) transformation. The test cohort was from the ICGC (https://dcc.icgc.org/). Samples were screened using the following criteria: 1) histological diagnosis of HCC, 2) available gene expression data, and 3) complete clinical data. The clinical features of the eligible patients are detailed in Table 1. Coincidentally, the case number for both the TCGA and ICGC cohorts was 231. The PRGs were obtained from the previous study (Supplementary Table S1) (Man and Kanneganti, 2015; Wang and Yin, 2017; Karki and Kanneganti, 2019; Xia et al., 2019; Ju A. et al., 2021; Shao et al., 2021). First, a differential expression analysis for the TCGA data was performed utilizing the “limma” R package according to the false discovery rate (FDR) < 0.05. We identified 40 genes (DEGs) differentially expressed between the tumor and normal samples. A univariate Cox analysis of overall survival (OS) was used to screen PRGs with prognostic value (*p* < 0.05). Overlapping PRGs with differential expression and prognostic value in the TCGA cohort were prepared for the construction of a prognostic model.

**TABLE 1:** Clinical features of the LIHC patients.

Features	TCGA (*n* = 231)		ICGC (*n* = 231)	
	Cases	%	Cases	%
Age (years)				
>65	70	30.3	142	61.5
≤65	161	69.7	89	38.5
Gender				
Male	159	68.8	170	73.6
Female	72	31.2	61	26.4
Grade				
G1	29	12.6	NA	NA
G2	101	43.7	NA	NA
G3	91	39.4	NA	NA
G4	10	4.3	NA	NA
T				
T1	115	49.8	NA	NA
T2	50	21.6	NA	NA
T3	56	24.2	NA	NA
T4	10	4.3	NA	NA
M				
M0	228	98.7	NA	NA
M1	3	1.3	NA	NA
N				
N0	227	98.3	NA	NA
N1	4	1.7	NA	NA
Stage				
Stage I	113	48.9	36	15.6
Stage II	49	21.2	105	45.5
Stage III	65	28.2	71	30.7
Stage IV	4	1.7	19	8.2

Grade: Edmonson tumor grade, Stage: tumor TNM, classification, T: tumor, M: distant metastasis, N: lymph node metastasis.

### Establishment and Verification of a Prognostic PRG Signature

A prognostic model was constructed with a LASSO-penalized Cox regression analysis to minimize the risk value of overfitting using overlapping PRGs besides CHMP3 that could not be detected in the ICGC dataset. In statistics and machine learning, the LASSO-penalized Cox regression is a useful method that identifies the most important elements to enhance the prediction accuracy of statistical model. LASSO is a popular machine learning algorithm, which was extensively utilized in medical studies (Liu et al., 2021a; Liu et al., 2021b; Liu et al., 2021c; Liu et al., 2021d). Thereafter, the calculation of the risk score was performed by the following formula: $Risk\;score=\sum_{i=1}^{n}\left(Coef_{i}*Exp_{i}\right)$ (Table 2). In our study, all patients were given a risk score and then classified into either high- or low-risk subgroups according to the median risk score of the TCGA cohort. A principal component analysis (PCA) based on the gene expression of the established signature was performed using the “stats” R package. The “survival” package, “survminer” package, and “timeROC” package in R were used to perform a KM survival analysis and receiver operating characteristic (ROC) analysis based on OS to estimate the prognostic precision of the gene signature in both sets.

**TABLE 2:** The corresponding coefficients of 9 PRGs in the risk model.

Gene	Coef
GSDMC	0.112
NOD2	0.207
CHMP4A	0.082
NLRP6	−0.205
CASP8	0.047
SCAF11	0.232
GPX4	0.318
CHMP6	0.067
CYCS	0.105

Coef, coefficient, Exp: the gene expression.

### Functional Enrichment Analysis

Gene Ontology (GO) and Kyoto Encyclopedia of Genes and Genomes (KEGG) signaling pathway enrichment analyses were performed for patients between the high-risk and low-risk subgroups utilizing the “clusterProfiler” R package. GO terms and KEGG signaling pathways with *p* < 0.05 showed statistical significance. The calculation of scores of about 16 immune cells and the activity of 13 immune-associated functions were performed according to single-sample gene set enrichment analysis (ssGSEA) in the “gsva” R package.

### Prognostic Independence of the Gene Signature From Conventional Clinical Features and Nomogram Generation

To further assess the independent prognostic value of the constructed gene signature, univariate and multivariate Cox regression analyses were utilized to determine whether it was influenced by other clinical characteristics, such as age, gender, grade, and TNM stage (Supplementary Tables S2, S3). A nomogram was drawn with the R package “RMS” on the basis of risk score and other clinical characteristics. A calibration curve was used for the evaluation of consistency between the predictive value and actual results.

### The Construction of ceRNA Network

A KM survival analysis based on OS in GEPIA (http://gepia.cancer-pku.cn/) was made for 9 genes in the model separately. CHMP4A, SCAF11, and GSDMC had significantly different results. According to the coexpression analysis and ENCORI online prediction tool (https://starbase.sysu.edu.cn/), eligible miRNAs were screened out. Moreover, a KM survival analysis was performed for eligible miRNAs. Only the analysis of miR-122-5p showed significantly different results. Furthermore, a coexpression analysis and ENCORI prediction of miR-122-5p were performed to identify eligible long non-coding RNAs (lncRNAs).

### Cell Cultures and RNA Interference

The human normal liver cell line HL-7702 and three liver cancer cell lines, HepG2, Hep3B, and SMMC7721, were prepared from the Cell Bank of the Chinese Academy of Sciences (Shanghai, China) and cultured in DMEM media with the supplementation of 10% fetal bovine serum (Gibco, United States). All of these cells were incubated at 37°C with 5% CO_2_. SiRNAs for SCAF11 were synthesized by RiboBio (Guangzhou, China). According to the manufacturer’s protocol, Lipofectamine 3000 (CA, United States) was mixed with siRNA to transfect liver cancer cell lines in a 6-well plate. The sequence of the negative control (si-NC) was not homologous to any human genomic sequence. The sequence of siRNA targeting SCAF11 was as follows: si-SCAF11-1 sense: 5′-CAT​GTC​CTA​TTG​ACC​GTA​AAC​CTT​T-3′; si-SCAF11-2 sense: 5′-CAT​TGG​AAG​GTT​ATG​TTA​AGG​TTC​A-3′.

### Quantitative Real-Time PCR

All RNA was extracted from cell lines using TRIzol (Invitrogen, United States). cDNA was synthesized from 1 μg RNA using the reagent Kit (Toyobo, Japan). Quantitative real-time PCR (qRT-PCR) was performed using the 7500 Fast quantitative PCR System (Applied Biosystems, USA). GAPDH was used for normalization of data, and these data were analyzed by 2^−ΔΔCT^. The primer sequences (Sangon Biotech, Shanghai, China) were as follows: SCAF11 forward primer, 5′-TGA​AAG​CAA​AGT​GTA​CCA​ACC​T-3′; SCAF11 reverse primer, 5′-GGC​TCT​CTA​TAA​GCT​CCT​CTG​T-3′; GAPDH forward primer, 5′-GTC​TCC​TCT​GAC​TTC​AAC​AGC​G-3′; GAPDH reverse primer, 5′-ACC​ACC​CTG​TTG​CTG​TAG​CCA​A-3′.

### Cell Proliferation, Migration, and Invasion Assays

Cell proliferation was detected through the Cell Counting Kit-8 assay (Beyotime Institute of Biotechnology, Shanghai, China) according to the manufacturer’s protocol. For the migration assay, 1 × 10^5^ cells were plated into the upper chamber with serum-free medium, and medium containing 10% fetal bovine serum was added into the lower chamber. After 48 h of incubation, the upper chamber cells were removed. The migrating cells were fixed with methanol and stained with 0.1% crystal violet. For the invasion assay, 50 μl of Matrigel was plated in the upper chamber, and the other procedures were the same as those for the migration assay. Migrated or invaded cells were imaged in a randomly chosen field of view and counted utilizing a ×200 microscope.

### Apoptosis Assay

According to the manufacturer’s protocol, apoptosis was detected using an Annexin-V-FITC apoptosis kit (BD Biosciences, Bedford, MD, United States). Flow cytometry was performed, and FlowJo 10 software (Tree Star Software, San Carlos, CA, United States) was used to analyze the results. The apoptosis rate was defined as the percentage of Q2 + Q3 cells.

### Statistical Analysis

R 4.0.4 and GraphPad Prism 7.1 were used for data analysis. The Mann–Whitney *U* test or Student’s *t*-test were used in the two-group analysis. Comparisons between multiple groups were performed by a Kruskal–Wallis one-way analysis of variance. *p* < 0.05 was considered to indicate statistically significant differences.

## Results

### Identification of Prognostic PRGs in the TCGA Cohort

**FIGURE 1:**
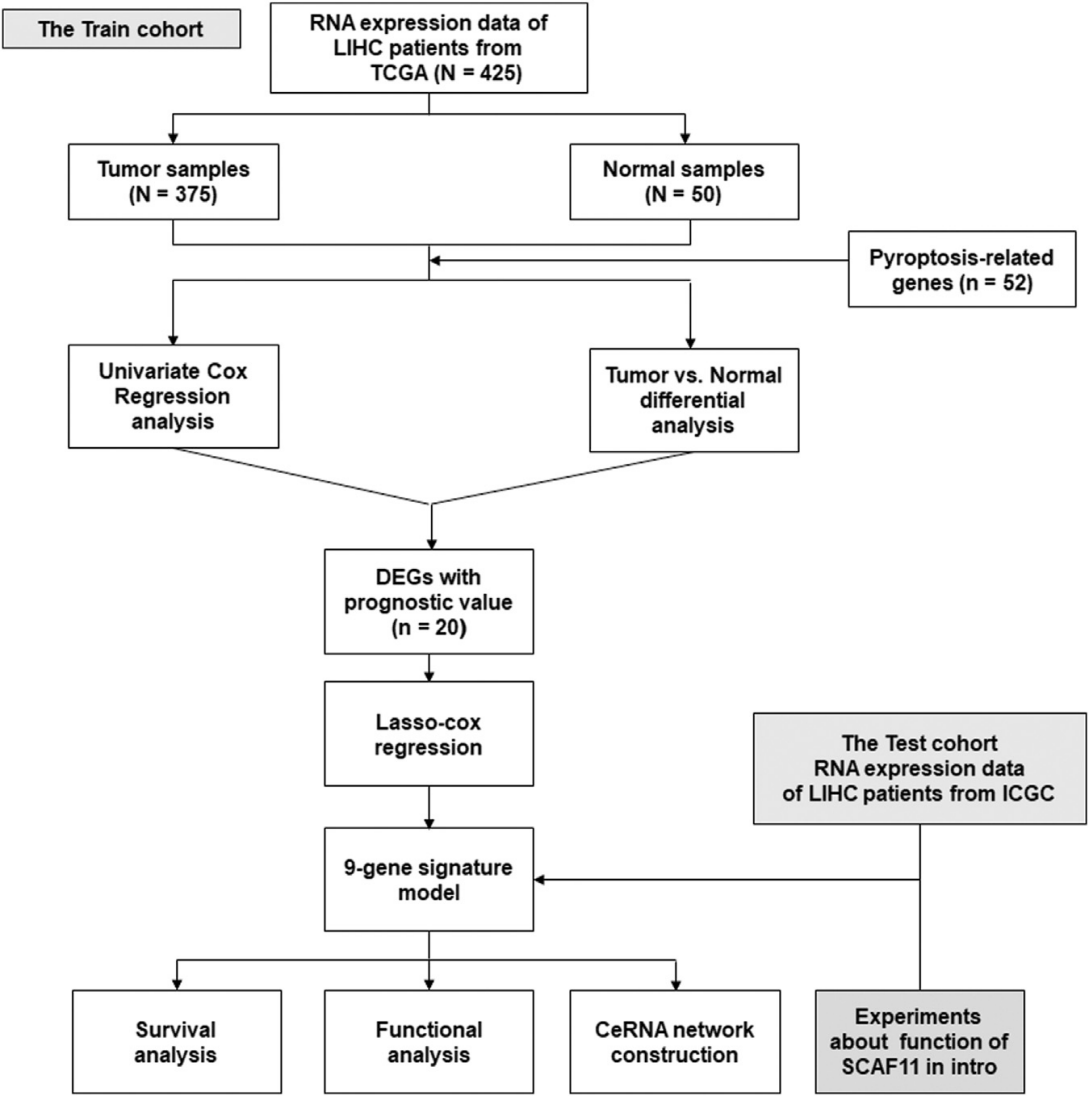
Flowchart of bioinformatics analysis.

**FIGURE 2:**
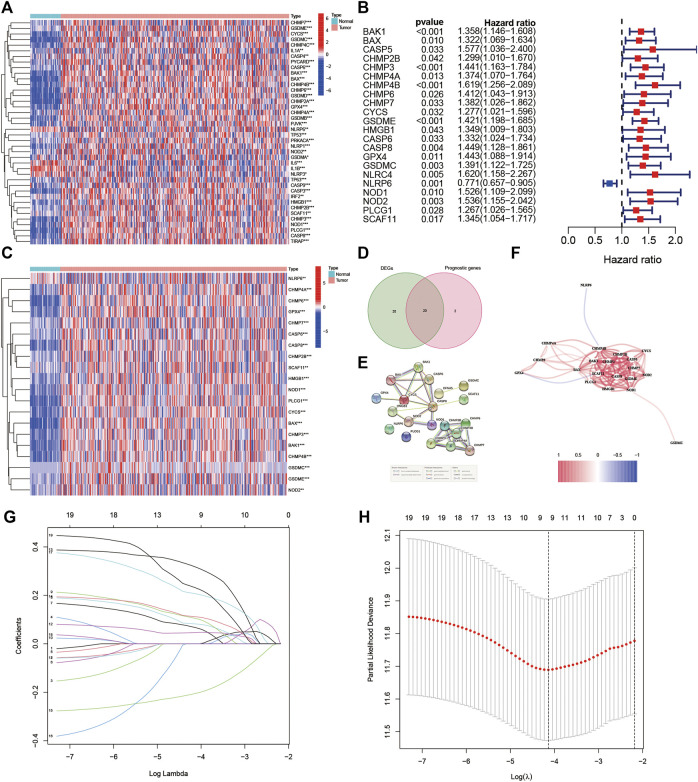
Identification of the prognostic pyroptosis-related DEGs in the TGCA cohort. **(A)** Heatmap displaying the pyroptosis-related DEGs. **(B)** Forest plots displaying the outcomes of the univariate Cox regression analysis between the expression of pyroptosis-related DEGs and OS. **(C)** Heatmap showing the overlapping genes. **(D)** Venn diagram showing the overlapping genes. **(E)** Protein–protein interaction network. **(F)** Association network of genes. **(G)** LASSO parameter profiles of the genes in the training cohort. **(H)** Parameter profile plot with the log(λ) sequence.

Figure 1 shows the flowchart. This research enrolled a total of 425 LIHC patients from the TCGA dataset. The general clinical information of those patients is shown in Table 1. As shown in Figure 1, LIHC samples from the TCGA-LIHC and the ICGC dataset were collected and analyzed. Interestingly, the case number for both the TCGA and ICGC cohorts was 231. We identified 40 DEGs (40/52, 76.9%) between LIHC samples and normal liver samples (Figure 2A). Twenty-two of 52 PRGs were considered OS-related in the univariate Cox regression analysis and were shown in the forest plot (Figure 2B). Overlapping PRGs with differential expression and OS-related prognostic value are shown in the heatmap and Venn diagram (Figures 2C,D). Moreover, the protein–protein interaction (PPI) online (https://string-db.org/cgi/input.pl) and correlation networks were visualized (Figures 2E,F). An OS-based LASSO Cox regression model was built using pyroptosis-related prognostic genes besides CHMP3 that could not be detected in ICGC (Figures 2G,H). When these 9 genes were enrolled, the model performed best (Table 2).

### Prognostic Analysis of the 9-Gene Signature in TCGA Dataset

**FIGURE 3:**
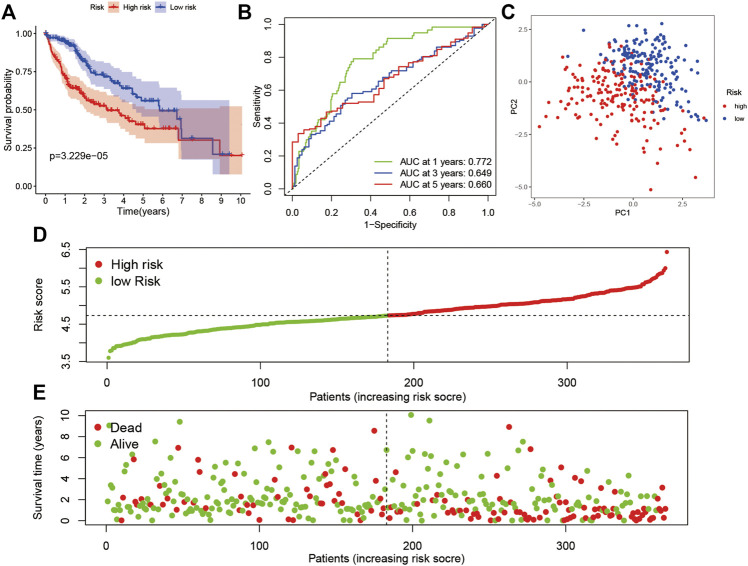
Prognostic analysis of the 9-gene signature in the TCGA cohort. **(A)** Survival analysis in the two risk subgroups. **(B)** AUC of the risk model. **(C)** PCA plot. **(D)** The risk score of LIHC in TCGA cohort. **(E)** Distribution of survival status.

To construct a pyroptosis-related risk model, a LASSO regression analysis was made based on the genes (GSDMC, NOD2, CHMP4A, NLRP6, CASP8, SCAF11, GPX4, CHMP6, and CYCS). To further evaluate the prognostic value and predictive performance of the risk model, KM survival and ROC analyses were made. The KM survival curve showed that patients in the high-risk subgroup had significantly worse survival results than the low-risk subgroup (Figure 3A; *p* < 0.001), and the AUC reached 0.772 at 1 year, 0.649 at 3 years, and 0.660 at 5 years in the ROC analysis (Figure 3B). All patients were classified into the high-risk subgroup or the low-risk subgroup and similarly distributed into two sets with PCA (Figure 3C). The distribution of risk score, survival status, and expression profile demonstrated that patients with high risk score had a lower possibility of surviving (Figures 3D,E).

### Verification of the Risk Model in the ICGC Dataset

**FIGURE 4:**
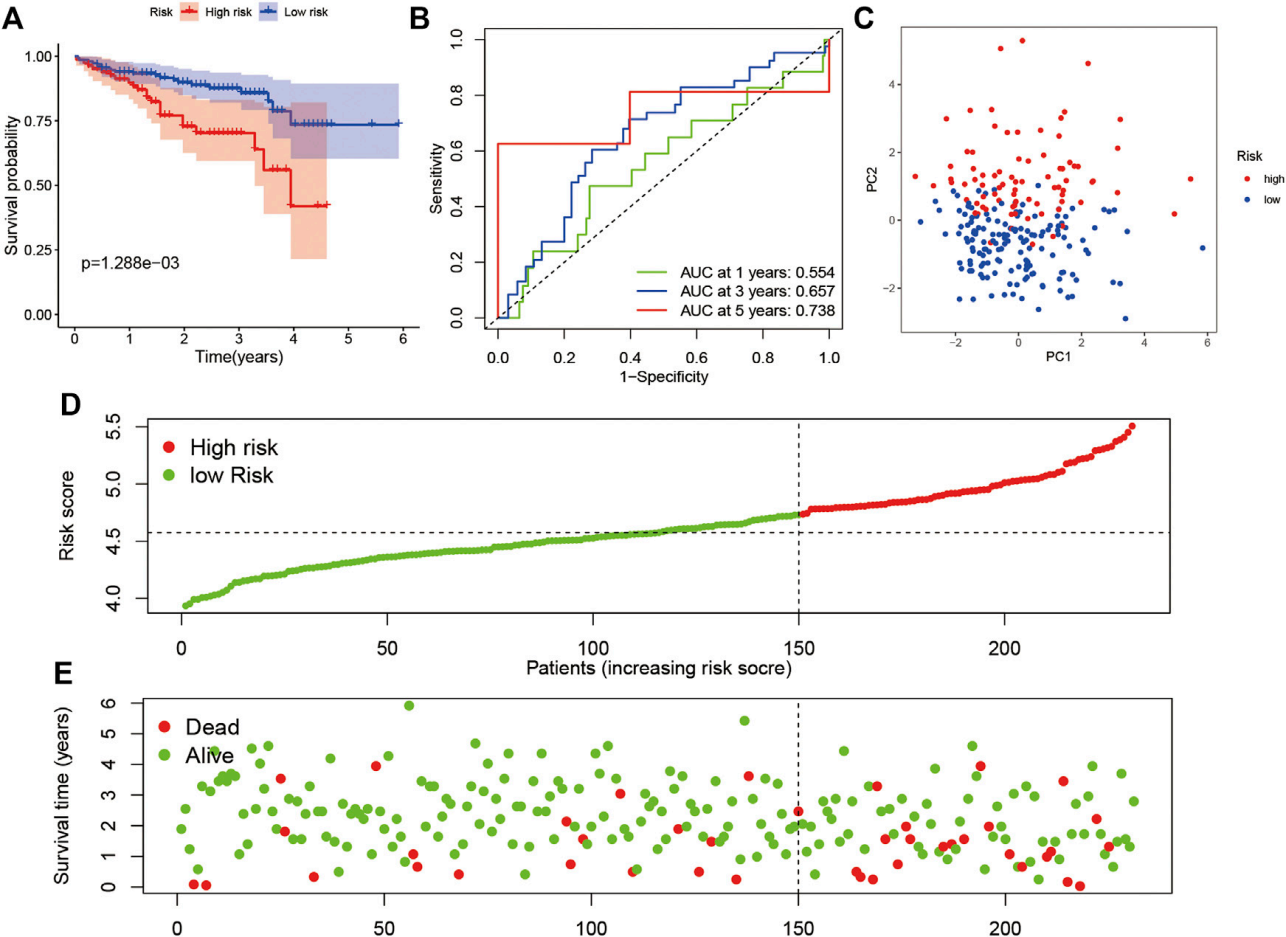
Verification of the risk model in the ICGC dataset. **(A)** Survival analysis in the two risk subgroups. **(B)** AUC of the risk model. **(C)** PCA plot. **(D)** The risk score of LIHC in the ICGC dataset. **(E)** Distribution of survival status.

According to the median value of the risk score in the TCGA cohort, we categorized patients in the ICGC cohort into either high-risk or low-risk subgroups. A KM survival analysis and ROC analysis were performed. Patients in the high-risk subgroup showed significantly worse OS than those in the low-risk subgroup (Figure 4A; *p* < 0.05). The AUC was 0.554 at 1 year, 0.657 at 3 years, and 0.738 at 5 years in the ROC analysis (Figure 4B). The PCA indicated that patients were divided into high- and low-risk subgroups (Figure 4C). The result of the risk curve was similar to the train set (Figures 4D,E).

### Multivariate Cox Regression Analysis and the 9-Gene Signature Risk Score ROC Analysis Based on Clinical Features

**FIGURE 5:**
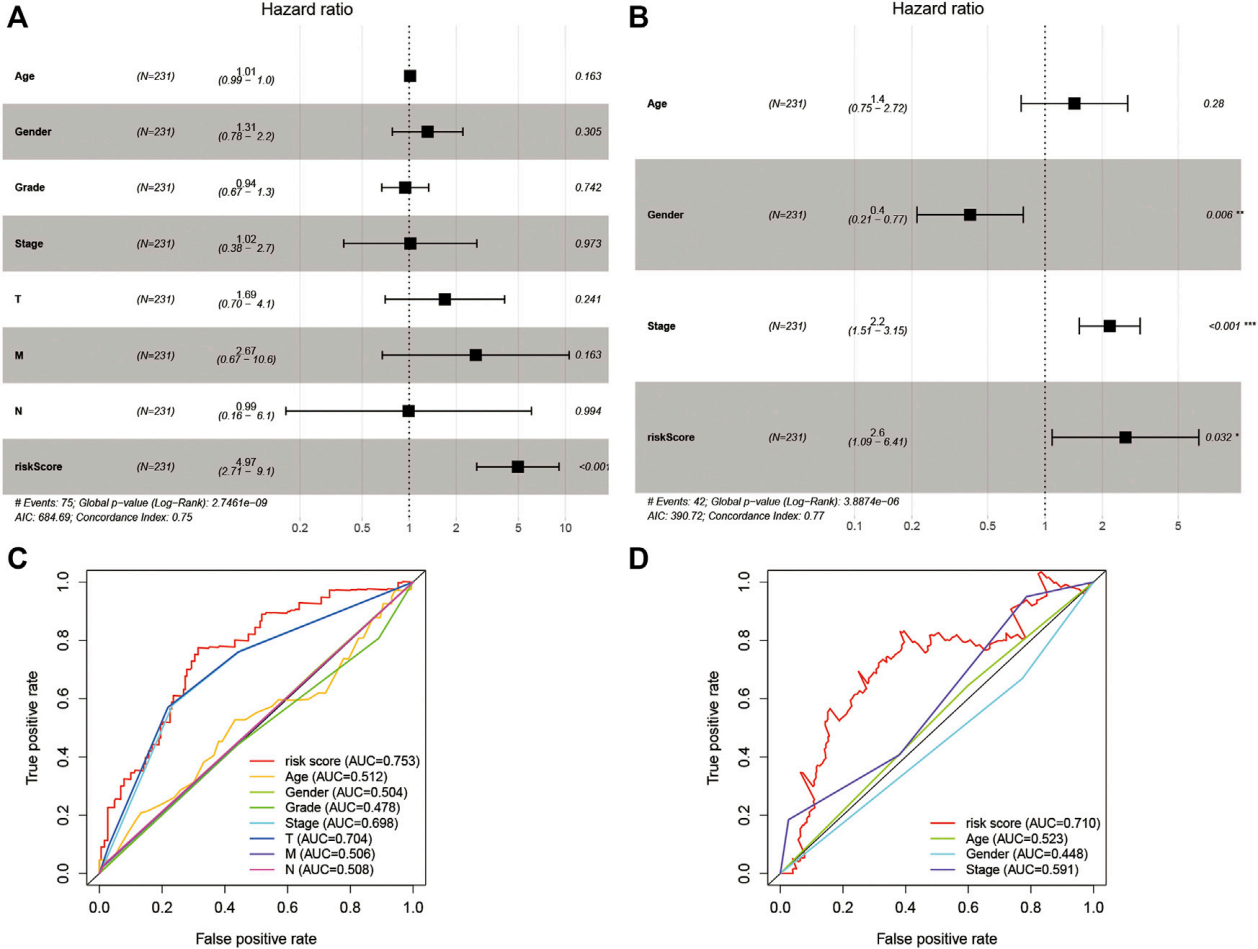
The results of the multivariate Cox regression analysis and the ROC analysis of the 9-gene signature risk score. **(A,B)** Multivariate Cox regression analysis including the 9-gene signature risk score and clinical features in the TCGA cohort and ICGC cohort based on OS. **(C,D)** ROC analysis based on the risk score and other clinical features. HR, hazard ratio. CI, confidence interval.

For evaluating the association of the risk score with the prognosis of the risk model, the risk score and clinical features were identified as indicators for univariate and multivariate Cox regression analysis in the TCGA and ICGC cohorts (Supplementary Table S4). The multivariate Cox regression analysis showed that the risk score was an independent prognostic factor for OS (Figures 5A,B; HR = 4.97, 95% CI = 2.71–9.10, *p* < 0.001 in TCGA; HR = 2.60, 95% CI = 1.09–6.41, *p* < 0.001 in ICGC). Subsequently, we performed an ROC analysis to evaluate how the 9-gene signature could act in the prediction of prognosis. The AUC reached 0.753 in TCGA and 0.710 in ICGC at 4 years and was superior to those of other clinical features (Figures 5C,D).

### Functional Enrichment Analysis

**FIGURE 6:**
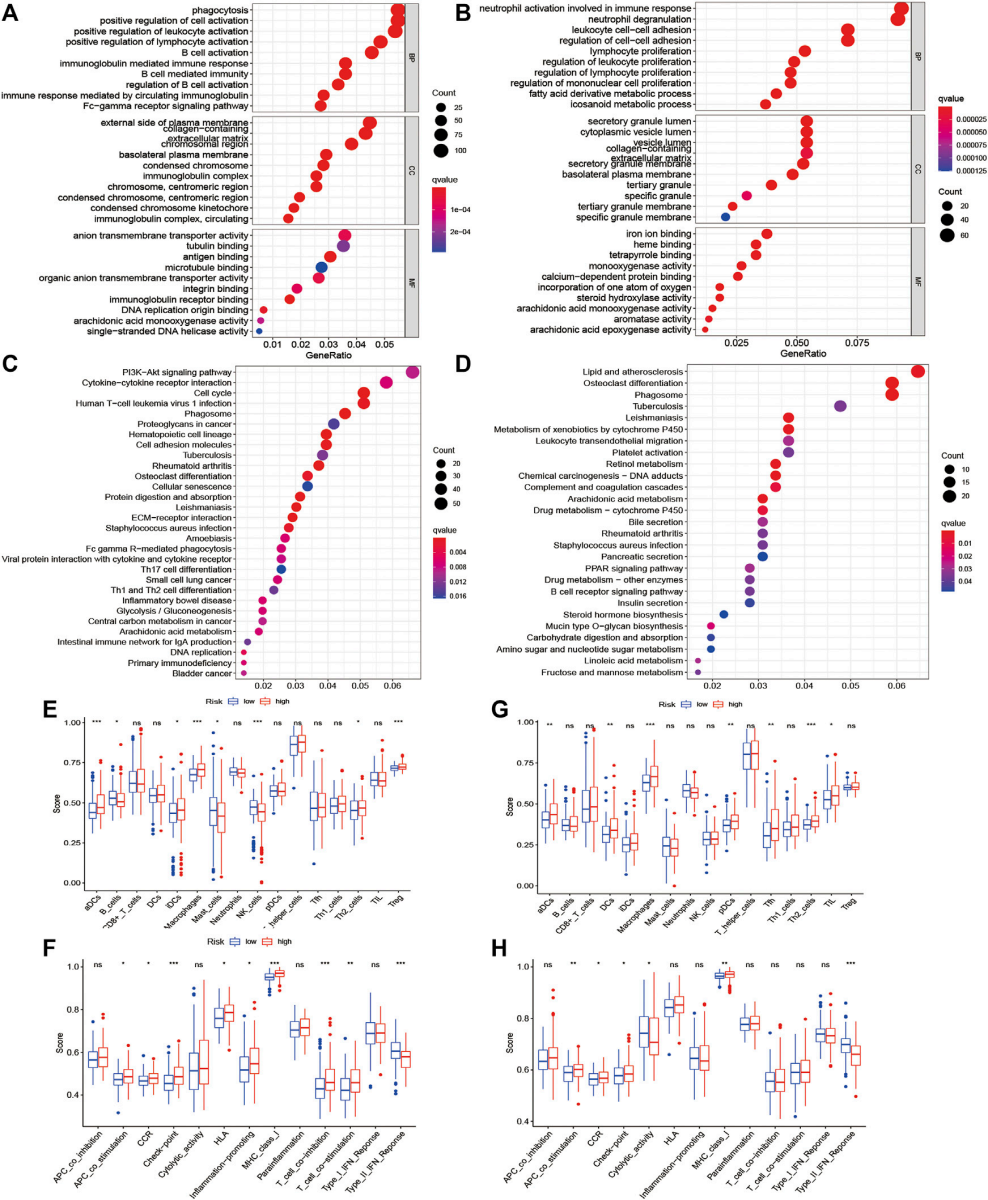
Functional enrichment analysis of two risk subgroups in the TCGA cohort and ICGC cohort. **(A–D)** GO enrichment and KEGG pathway analyses in the TCGA cohort and ICGC cohort. **(E–H)** Levels of diverse immune cell subpopulations and immune-related functions in the TCGA cohort and ICGC cohort.

The functional roles of DEGs between the high-risk and low-risk subgroups were explored by GO and KEGG enrichment analyses. The GO analysis results showed that DEGs were mainly enriched in phagocytosis, regulation, and activation of immune cells, and some metabolic process signaling pathways (Figures 6A,B). Moreover, the KEGG analysis showed the significant enrichment of genes in PI3K-Akt signaling and lipid and atherosclerosis (Figures 6C,D). To further explore the immune states in the two risk subgroups, the infiltrating levels of various immune cell subpopulations and immune-associated features were quantified with ssGSEA. For immune cells, the infiltrating levels of activated dendritic cells, B cells, immature dendritic cells, macrophages, mast cells, NK cells, Th2 cells, and regulatory T cells were greatly different between the low-risk and high-risk subgroups in the TCGA cohort (Figure 6E). In addition, there were significant differences in immune function levels, including antigen-presenting cell costimulation, chemokine receptor, checkpoint, HLA, inflammation-promoting, and other immune-related functions, between the low-risk and high-risk subgroups (Figure 6F). There was also a remarkable difference between the two subgroups in the ICGC cohort (Figures 6G,H).

### Construction of Survival Prediction Nomogram

**FIGURE 7:**
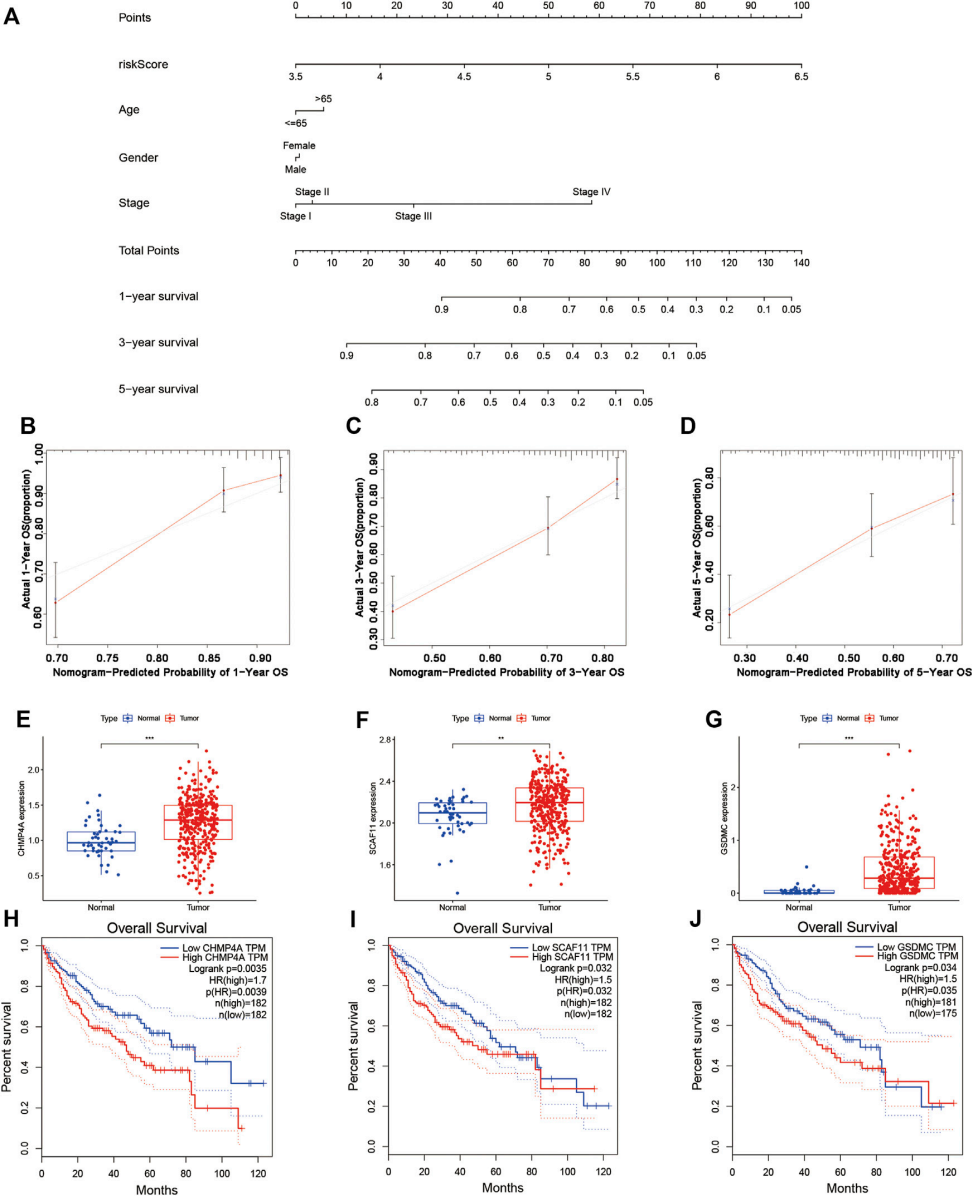
Establishment of the survival prediction nomogram and calibration plot of the nomogram. **(A–D)** The nomogram and calibration plot in TCGA cohort. **(E–G)** Analysis of gene expression in the risk model. **(H–J)** KM survival analysis based on OS in the two subgroups.

In the TCGA cohort, we established a nomogram based on the risk score and clinical features of each patient for predicting the 1-year, 3-year, and 5-year survival rates (Figure 7A). The points in the nomogram represented the contribution of the risk score and clinical features to OS. By adding all points of each factor to calculate the total points, the corresponding survival rates at 1 year, 3 years, and 5 years were obtained. The corresponding calibration curves suggested that the OS calculated by the nomogram agreed with the actual OS (Figures 7B–D).

### KM Survival Analysis for 9 Genes in the Risk Model and Coexpression Analysis

A KM survival analysis based on OS in GEPIA was performed for 9 genes in the model separately. CHMP4A, SCAF11, and GSDMC had significantly different results. The expression of CHMP4A, SCAF11, and GSDMC was high in liver cancer tissue (Figures 7E–G). Patients in the low-expression group had greatly better OS than those in the high-expression group (Figures 7H–J). According to the coexpression analysis (R < −0.2, *p* < 0.001) and ENCORI online analysis for CHMP4A, SCAF11, and GSDMC, we found that miR-885-5p and miR-122-5p were eligible and may interact with SCAF11 (Supplementary Figures S1A–D). A KM survival analysis based on OS was performed for miR-885-5p and miR-122-5p. Patients in the low-expression miR-122-5p group had greatly worse OS than those in the high-expression group (Supplementary Figure S1F; *p* < 0.05). Furthermore, a coexpression analysis (*R* < −0.2, *p* < 0.001 for miR-122-5p) and ENCORI prediction were performed to identify lncRNAs. NUTM2B-AS1, LINC01278, GUSBP11, LINC00205, LINC00294, PCBP1-AS1, AP4B1-AS1, SNHG7, LINC01140, and MMP25-AS1 had significant correlations (Supplementary Figure S2). Finally, we constructed a ceRNA network composed of SCAF11 mRNAs, miR-122-5p, and 10 lncRNAs (Supplementary Figure S3).

### The Expression of SCAF11 Was Upregulated in HCC

**FIGURE 8:**
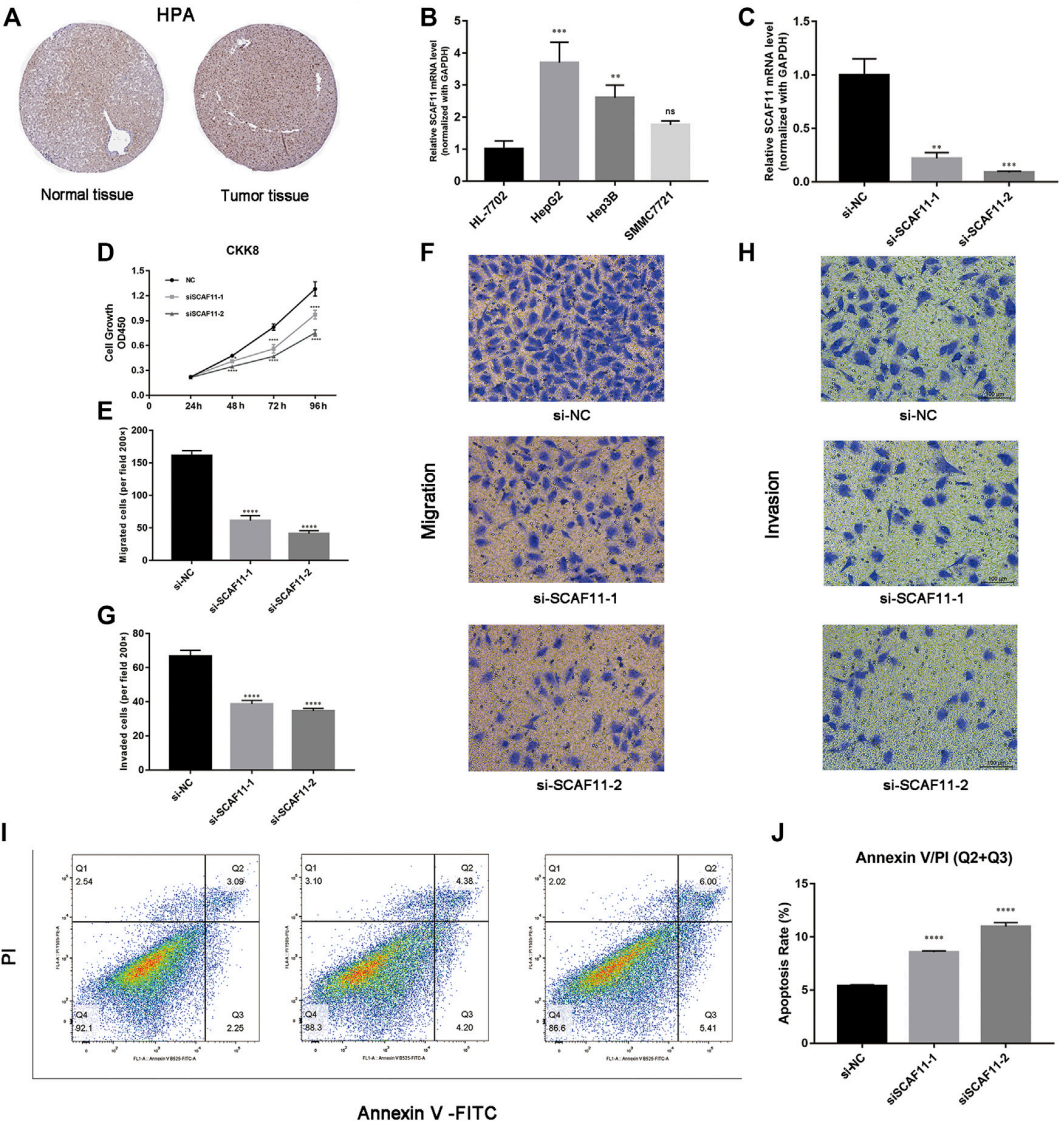
SCAF11 promoted proliferation, migration, and invasion and suppressed apoptosis of HCC cell lines. **(A,B)** The HPA and qRT–PCR results showed that SCAF11 was upregulated in HCC cell lines. **(C)** SCAF11 was inhibited by siRNAs. **(D)** Cell proliferation assay. **(E–H)** Analysis of the effect of SCAF11 on the migration and invasion of HCC cells. **(I,J)** Analysis of the effect of SCAF11 on HCC cell apoptosis by flow cytometry. **p < 0.01; ***p < 0.001; ****p < 0.0001.

Based on the core role of SCAF11 in ceRNA, we detected the expression of SCAF11 analyzed by immunohistochemical staining between normal and tumor liver tissue in the Human Protein Atlas (HPA; https://www.proteinatlas.org/). We found that the expression of SCAF11 was roughly upregulated in liver cancer tissue (Figure 8A). Then, we found that the mRNA expression of SCAF11 was higher in HCC cell lines than in the nontumorigenic cell line HL-7702 (Figure 8B). To further explore the function of SCAF11, HepG2 cell lines were chosen for subsequent experiments. Two different siRNAs were designed and transfected into HepG2 cells to knock down SCAF11. The qRT–PCR results showed that the mRNA expression of SCAF11 was downregulated by the siRNAs (Figure 8C).

### Downregulation of SCAF11 Affected Proliferation, Migration, Invasion, and Apoptosis of HCC Cells

The results of the above analysis suggested that SCAF11 was overexpressed in HCC, so we further explored its function in cell experiments. The results of downregulated SCAF11 could effectively inhibit HepG2 cell proliferation (Figure 8D). Transwell assays were used to investigate the effects of SCAF11 knockdown on the invasion and migration capability of HCC cells. The results showed that fewer HepG2 cells migrated in the siRNA group than in the si-NC group (Figures 8E–H), indicating that SCAF11 knockdown could inhibit the migration and invasion capacities of HCC cells. Furthermore, we performed flow cytometry to detect apoptosis in HCC cell lines. The results showed that compared with HCC cells in the si-NC group, those in the siRNA-treated group had a significantly increased apoptotic ratio (Figures 8I,J).

## Discussion

As a highly inflammatory form of programmed cell death, pyroptosis has features such as cellular membrane pore generation, cytoplasmic swelling, membrane rupture, and the secretion of IL-18 and IL-1β (Bergsbaken et al., 2009; Fang et al., 2020). Due to its inflammatory effects, pyroptosis is thought to be specific to both immune normal cells and nonimmune cells. Overactivated pyroptosis can cause an abnormal inflammatory response (Hage et al., 2019; Hou et al., 2020). There are several studies about the relationship between pyroptosis and a variety of diseases, especially inflammatory diseases and cancers (Jiang et al., 2020; Zheng et al., 2020). Researchers have been considering the induction of pyroptosis as a new effective treatment for cancers. A study has reported that metformin activates the NF-κB signaling pathway to drive caspase-3/GSDME-mediated pyroptosis in cancer cells (Jiang et al., 2020). In pancreatic ductal adenocarcinoma, mammalian STE20-like kinase 1 can induce ROS-induced pyroptosis, promoting cancer cell death and suppressing proliferation, migration, invasion, and cell spheroid formation (Cui et al., 2019). However, there are few studies about pyroptosis in HCC. In recent years, many novel biomarkers have been found to predict patient prognosis, but this is the first time to establish a prognostic model related to pyroptosis in HCC patients. We found that 40 PRGs were differentially expressed between normal tissues and liver tumor tissues. A new risk model that was established by LASSO Cox regression analysis was verified in the ICGC dataset.

The risk model established was composed of 9 PRGs: GSDMC, NOD2, CHMP4A, NLRP6, CASP8, SCAF11, GPX4, CHMP6, and CYCS. GSDMC is a member of the GSDM family, and its biological and cytological function has not been clearly explored (Wei et al., 2020). A recent study reported that when GSDMC is specifically cleaved by caspase-8 following TNF-α stimulation, it can generate the N-terminal domain that forms pores on the cell membrane and induces pyroptosis, leading to necrosis (Feng et al., 2018; Hou et al., 2020). NOD2 is a member of the Nod1/Apaf-1 family with two caspase recruitment (CARD) domains and six leucine-rich repeats (LRRs). It exerts an effect on the immune response to intracellular bacterial lipopolysaccharides (LPS) by identifying muramyl dipeptide (MDP) and activating the NF-κB signaling pathway (Shi et al., 2020). Pyroptosis can be caused by histone H3 through the NOD2-RIP2 and VSIG4 signaling pathways (Shi et al., 2020). As the sensor component of the NLRP6 inflammasome, NLRP6 exerts an important effect on innate immunity and inflammation (Liu et al., 2018). NLRP6 could induce pyroptosis to decrease neuronal viability (Zhang J. et al., 2020). CASP8 is a member of the cysteine-aspartic acid protease (caspase) family. Caspase-8 controls the balance of apoptosis, necroptosis, and pyroptosis at later stages of embryonic development (Fritsch et al., 2019). GPX4 belongs to a family of phylogenetically related enzymes and is upregulated at the protein level in HCC (Guerriero et al., 2015). The study has revealed that GPX4 is upregulated across cancers and has the function of promoting the tumor progression (Zhang X. et al., 2020). CHMP6 is a member of the chromatin-modifying protein family. Its function in tumors is still unknown. Meanwhile, CYCS expression levels have been found to be upregulated in triple-negative breast cancer, and its effects have rarely been studied (Lin et al., 2021). SCAF11 (SR-related CTD associated factor 11), also known as CASP11, is an intracellular receptor for LPS and regulates pyroptosis (Abdalla et al., 2012; Hagar et al., 2013; Yang et al., 2015; Chen et al., 2019; Agnew et al., 2021). The study has showed that noncanonical CASP11 activation activates GSDMD to cause pyroptosis in alcoholic hepatitis and worsen hepatocellular lytic death (Khanova et al., 2018). In our study, SCAF11 was the only hub gene among the PRG signatures in the ceRNA network. According to the coexpression analysis and ENCORI online analysis, SCAF11 might interact with miR-122-5p in LIHC. Furthermore, loss-of-function experiments for SCAF11 *in vitro* were performed in HCC cell lines for the first time. Although some of these genes have been studied in pyroptosis, they have been combined as a marker of prognosis for LIHC patients for the first time.

This research utilized two completely independent datasets and developed a PRG signature to predict the OS of patients with LIHC. The risk model established was made up of 9 PRGs. Patients were classified into high-risk and low-risk subgroups based on their median risk score in the TCGA cohort. The KM survival curve suggested that patients in the high-risk subgroup showed significantly worse survival results than patients in the low-risk subgroup. The prognostic reliability of this signature was evaluated with ROC curve analysis. In addition, we visualized the results of multivariate Cox regression analysis using a forest plot with risk score and other different clinical features, and the results showed that the risk score was an independent prognostic index in both the TCGA cohort and ICGC cohort. Further GO and KEGG enrichment analyses found that DEGs were mainly enriched in phagocytosis, regulation, and activation of immune cells, and some metabolic process signaling pathways, indicating that the immune microenvironment may play an important role in HCC progression. Moreover, ssGSEA suggested obvious differences in the immune cell subpopulations and immune-related features between the high-risk subgroup and low-risk subgroup, such as activated dendritic cells, B cells, immature dendritic cells, macrophages, mast cells, NK cells, Th2 cells, regulatory T cells, antigen-presenting cell costimulation, chemokine receptor, checkpoint, HLA, inflammation-promoting, and other immune-related functions. Similarly, it may suggest that immune cells and immune function are involved in the progression of liver cancer. The nomogram results suggested that the risk model was an efficient approach for predicting the OS of LIHC patients, and the corresponding calibration curves showed good consistency. Moreover, the ceRNA network was constructed with PRGs in LIHC. Combined with experiments, SCAF11 was found to be upregulated in HCC.

Downregulation of SCAF11 inhibited the proliferation, migration, and invasion and promoted the apoptosis of HCC cells.

## Conclusion

In summary, a prognostic signature of 9 PRGs in LIHC patients (GSDMC, NOD2, CHMP4A, NLRP6, CASP8, SCAF11, GPX4, CHMP6, and CYCS) was developed. Furthermore, the model had good performance and was validated by the TCGA and ICGC databases. At the same time, the ceRNA network was constructed with PRGs in LIHC. In addition, our experiments confirmed that SCAF11 acted as an oncogene that might promote progression of liver cancer.

## Data Availability

The original contributions presented in the study are included in the article/Supplementary Material, further inquiries can be directed to the corresponding author.

## Supplementary Material

Supplementary Figure 1 MiRNAs of the coexpression analysis and survival analysis. **(A,B)** Coexpression analysis between the expression of SCAF11 and miR-885-5p or miR-122-5p. **(C,D)** Analysis of miR-885-5p and miR-122-5p expression. **(E,F)** KM survival curves for miRNAs in the TCGA cohort.

Supplementary Figure 2 Coexpression analysis between miR-122-5p and lncRNAs and expression analysis of lncRNAs. **(A)** The coexpression analysis between miR-122-5p and lncRNAs. **(B)** The expression analysis of lnRNAs. **(C)** The coexpression analysis between SCAF11 and lnRNAs.

Supplementary Figure 3 The construction of a ceRNA network based on genes in the pyroptosis-related hepatocellular carcinoma model.

Supplementary Table 1 Pyroptosis-associated genes.

Supplementary Table 2 TCGA clinical information.

Supplementary Table 3 ICGC clinical information.

Supplementary Table 4 Univariate and multivariate Cox regression analyses.
