# Supplementary figures and images for: Identification of a Pyroptosis-Related Prognostic Signature Combined With Experiments in Hepatocellular Carcinoma
Source: Front Mol Biosci. 2022 Mar 4;9:822503. doi: 10.3389/fmolb.2022.822503 (PMC8931679; doi:10.3389/fmolb.2022.822503)

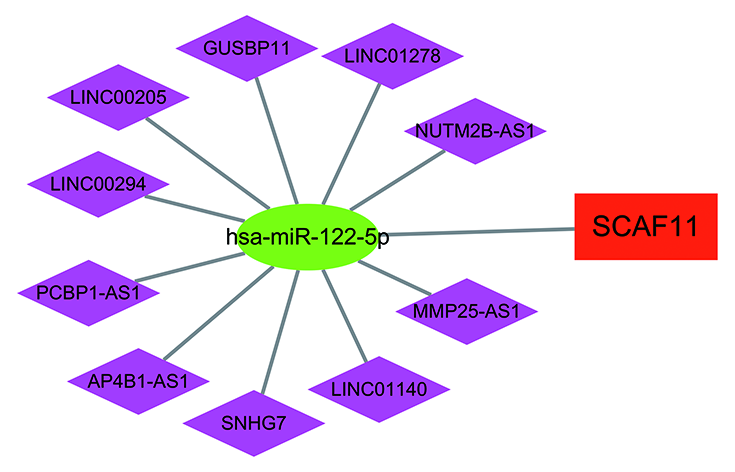

Supplement: Supplementary file 3 [file Image3.TIF]

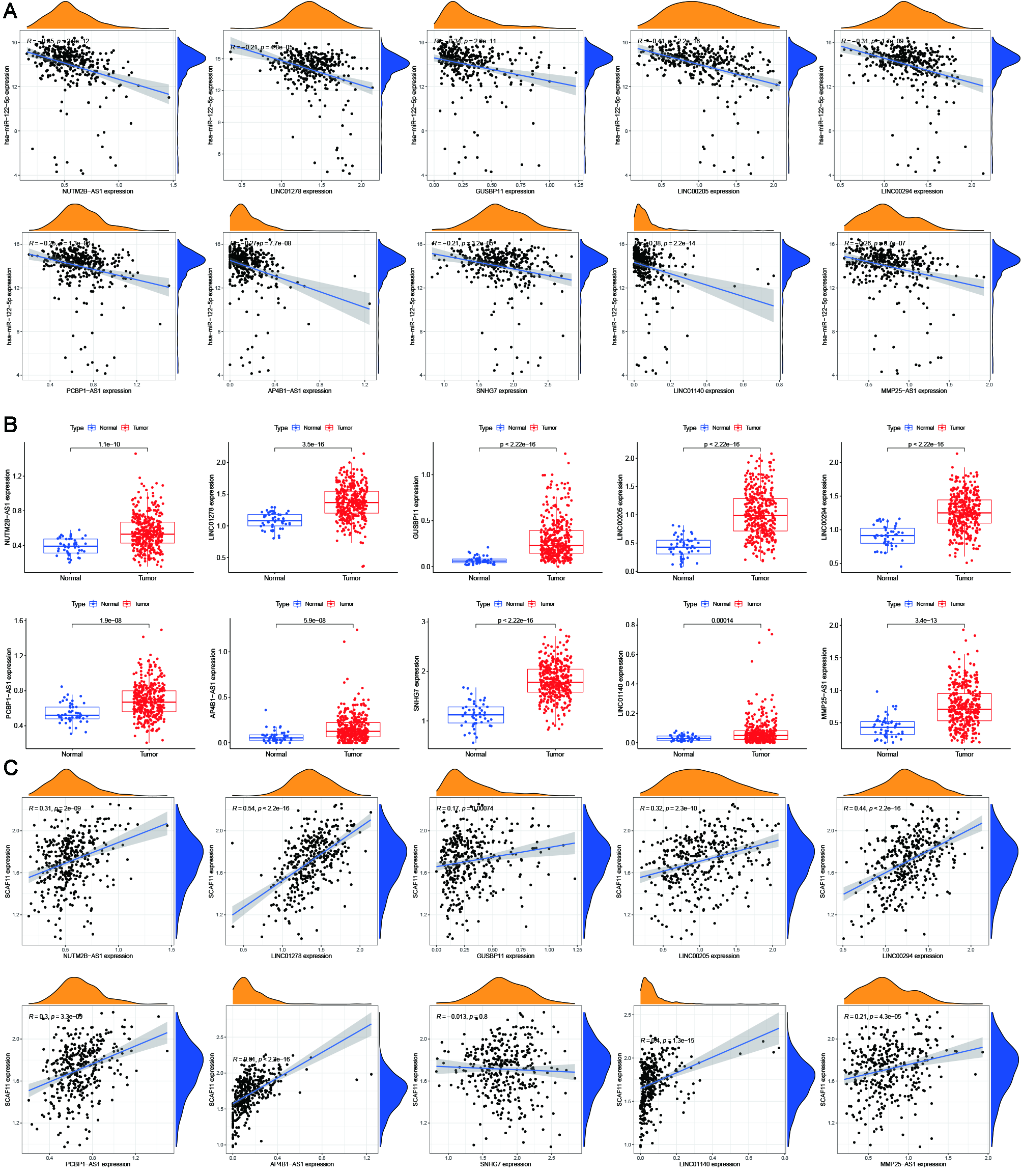

Supplement: Supplementary file 4 [file Image2.TIF]

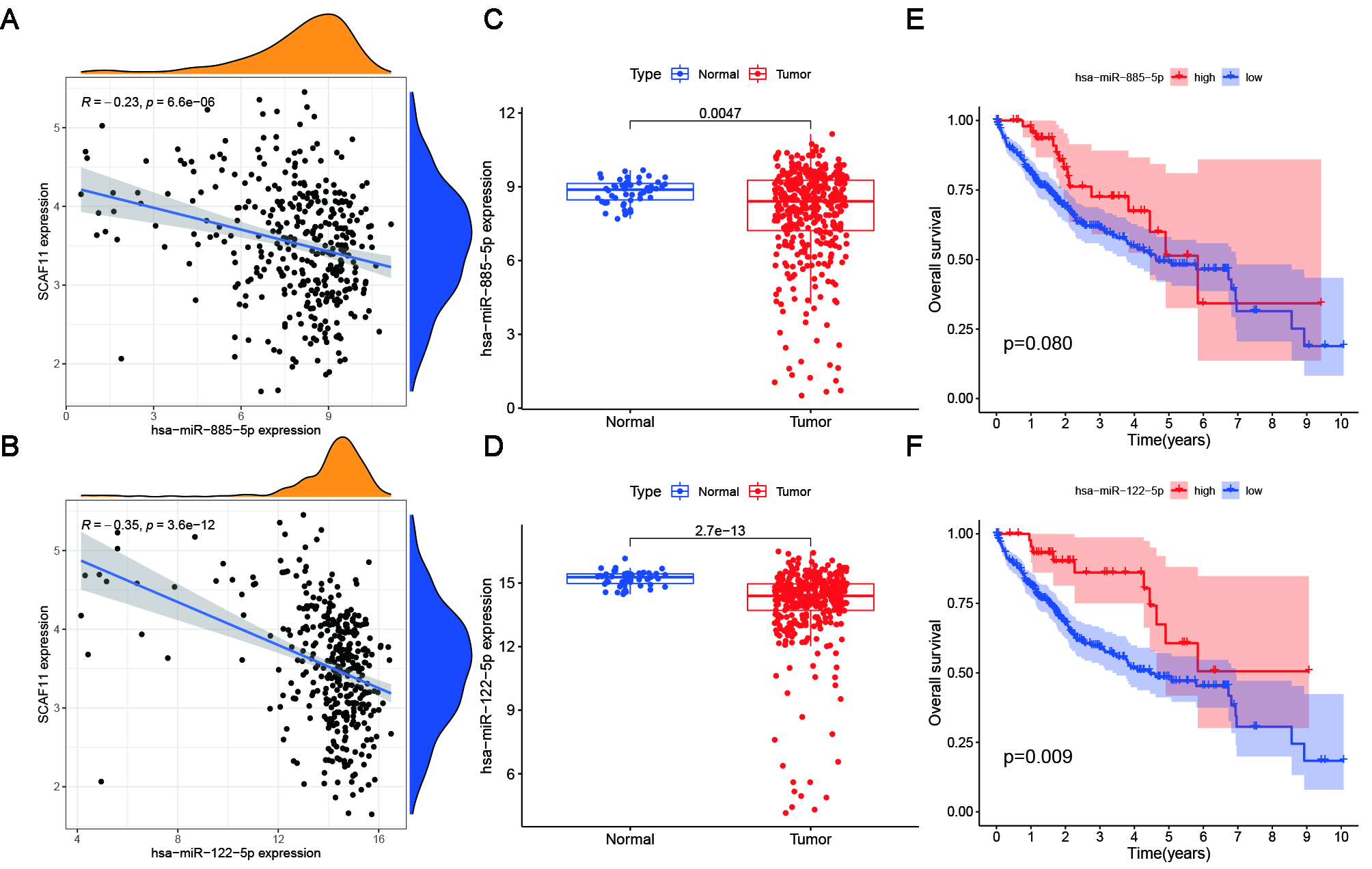

Supplement: Supplementary file 5 [file Image1.TIF]
